# Functional differences between Andean oak (*Quercus humboldtii* Bonpl.) populations: The importance of intraspecific variation

**DOI:** 10.1371/journal.pone.0299645

**Published:** 2024-03-13

**Authors:** Diana Bonilla, Valentina Castañeda-León, Adriana Corrales, Ana M. Aldana, Julieth V. Cárdenas, Adriana Sanchez

**Affiliations:** 1 Biology Department, Faculty of Natural Sciences, Universidad del Rosario, Bogotá, D.C., Colombia; 2 Departamento de Ciencias Biológicas, Universidad de Los Andes, Bogotá, D.C., Colombia; 3 Santuario de Fauna y Flora Guanentá Alto Río Fonce, Parques Nacionales Naturales de Colombia, Bogotá, D.C., Colombia; University of Udine: Universita degli Studi di Udine, ITALY

## Abstract

Monodominant tree communities can have phenotypic trait variation (intraspecific variation) as extreme as the trait variation across a forest with higher species diversity. An example of such forests is those composed of *Quercus*, an important genus of woody angiosperms in the montane neotropical forest. The Andean oak, or *Quercus humboldtii* Bonpl., is the sole member of this genus in South America and a characteristic component of montane ecosystems. Although there are several studies on the ecology and genetic structure of this species, there are few studies on the functional trait diversity among populations. Understanding functional traits can improve our comprehension of how organisms respond to various environmental conditions. In this study, we aimed to evaluate differences in six functional traits in individuals of the Andean oak, in two ontogenetic stages (juveniles and adults) from three populations with contrasting environmental conditions. Additionally, using T-statistics, we assessed the impact of external filters (e.g., climate, resource availability, large-scale biotic interactions) on population assembly. We found a remarkable level of functional differentiation among Andean oak forests, with all traits differing between populations and five traits differing between ontogenetic stages. External filters had a stronger influence in populations with more extreme environmental conditions. These findings emphasize the dynamic and context-dependent nature of functional traits in this species. However, given the limited exploration of functional diversity in Andean oak populations, further studies are needed to inform conservation efforts.

## Introduction

When thinking about tropical ecosystems, rainforests with highly diverse plant communities and low relative abundance typically come to mind. However, exceptions to this general pattern exist throughout the tropics, where a single tree species can account for more than 60% of the total abundance [1, 2]. While monodominant forests are rare, their community-level phenotypic variation can be as extreme as that observed in forests with higher species diversity [3]. As a result, intraspecific variation within these forests may be as crucial as variation among species for community structure and ecosystem function [4, 5].

An example of such monodominant forests is those composed by *Quercus* L. *Quercus* is a remarkable group of woody flowering plants in the Neotropics, ranging from central Mexico to the northern Andes [6]. The highest species diversity for this genus in the Americas is found in the mountains of southern Mexico [7]. However, as we move towards Central America, there is a gradual decrease in species diversity to the extent that only one oak species, *Q*. *humboldtii* Bonpl. (1805), exists in Colombia. Although *Q*. *humboldtii* is a relatively recent migrant in this country [8], it remains a distinctive element of its montane ecosystems [9]. This species forms monodominant forests [10] that span the three branches (Eastern, Central, and Western Cordillera) of the Colombian Andes, occurring at elevations between 750 and 3450 meters [11, 12].

Despite their monodominance, Andean oak forests harbor significant levels of biodiversity and serve as refuge and critical habitat for various species of plants, animals, and fungi [13]. These forests, known as "robledales" in Spanish, are part of the Andes biodiversity hotspot [14] and provide essential ecological services, including habitat protection, water provision, and climate regulation [15]. However, despite all these benefits, they remain poorly known among the population and have been subjected to high rates of deforestation due to timber extraction and expansion of agricultural activities [16]. Therefore, studying this species and understanding the factors that determine the assemblage of its communities is crucial.

An interesting aspect of this species is its wide altitudinal distribution, which implies a broad climatic adaptation [8, 9]. The altitudinal gradient inhabited by *Q*. *humboldtii* encompasses temperatures ranging from 9.3 to 27.9°C, with a mean temperature of approximately 20°C, and precipitation varying from around 788 mm/year in dry environments to 2681 mm/year in very humid regions. Although several studies have been conducted on this species, few have attempted to explain its wide climatic adaptation from a functional perspective [e.g., 17, 18], which could be key to understanding their ample distribution. Plant functional traits are well-defined heritable characteristics that influence fitness [19], encompassing morphological, physiological, or phenological features. These traits have been widely used to investigate species-environment relationships and are linked to factors such as temperature and precipitation [19–21]. Therefore, we hypothesize that, as a strategy to adapt to a wide range of climatic conditions, *Q*. *humboldtii* may exhibit broad functional differentiation driven by local conditions. This hypothesis of functional traits differentiation could be supported by previous studies, including the high genetic diversity identified by Zorrilla-Azcué et al. [9] in *Q*. *humboldtii* populations, as well as the historical controversy surrounding the number of *Quercus* species in Colombia, with up to six different candidate species [8].

In a climatic gradient, we can expect distinct patterns in plant functional traits. In regions with higher precipitation and favorable temperature conditions, traits such as leaf area (LA), specific leaf area (SLA), and specific root length (SRL) are likely to increase [20, 22]. This reflects a strategy of maximizing resource acquisition and growth in environments with abundant water and suitable temperatures [19]. Conversely, in areas with lower precipitation and extreme temperature conditions, traits such as leaf thickness (LT), leaf dry matter content (LDMC), and wood density (WD) are expected to increase [23, 24]. These traits indicate an adaptation towards water conservation and structural integrity in environments where water availability is limited, and temperature stress is prevalent [19]. However, it is important to note that these trait patterns are generalizations and that other factors such as soil conditions, facilitation or trade-offs between traits may modify plant trait responses [25].

Ontogeny is another potential factor underlying the functional differentiation in plants. While conspecific individuals are commonly considered ecologically equivalent, evidence suggests there is significant variation in specialization among populations, carrying important ecological, evolutionary, and conservation implications [26]. Specifically, in the context of Andean oak forests, the coexistence of functionally distinct adult and juvenile individuals could favor climatic adaptation through temporal niche partitioning, known as the storage effect [27, 28]. This mechanism would optimize resource utilization and improve the ability of *Q*. *humboldtii* to thrive in diverse environmental conditions. However, to our knowledge, this hypothesis remains unexplored.

In addition to examining plant functional trait patterns, it is crucial to assess the influence of climate and other factors on community assembly processes. In this regard, the framework proposed by Violle et al. [29] offers a unique approach that focuses on individual-level, rather than species-level analysis, which is particularly valuable when studying monodominant forests. By adopting this framework, we can investigate the impact of external filters on the traits of *Q*. *humboldtii* and the composition of its populations. External filters encompass a range of environmental factors and processes that operate at scales larger than the community, including climatic conditions, soil characteristics, disturbances, and biotic interactions. These filters exert selective pressures that can either facilitate or constrain specific traits, resulting in non-random trait distributions within communities. Within the specific context of *Q*. *humboldtii*, we hypothesize that populations inhabiting more extreme climatic conditions, such as those characterized by cold or drought, will experience an amplified influence of external filters. Moreover, we anticipate that the magnitude of this influence will differ significantly between the adult and juvenile stages of the species, reflecting variations in their respective adaptive strategies and responses to environmental constraints.

In summary, the objective of this study is to understand how functional traits of the Andean oak, *Quercus humboldtii*, change between populations and two ontogenetic stages (juvenile and adult). We expect intraspecific trait variability to be different between populations with contrasting environments (Hypothesis 1; H1) and between ontogenetic stages in the same population (adults different from juveniles; Hypothesis 2; H2). Moreover, we aim to explore the role of external filters, such as climate, in determining the population composition of *Q*. *humboldtii* along the climatic gradient. We hypothesize that, consistent with the expected functional differentiation between populations and ontogenetic stages, the strength of the filters will also change, with more pronounced effects on populations living in more extreme climatic conditions (Hypothesis 3; H3).

## Materials and methods

### Study area

**Fig 1:**
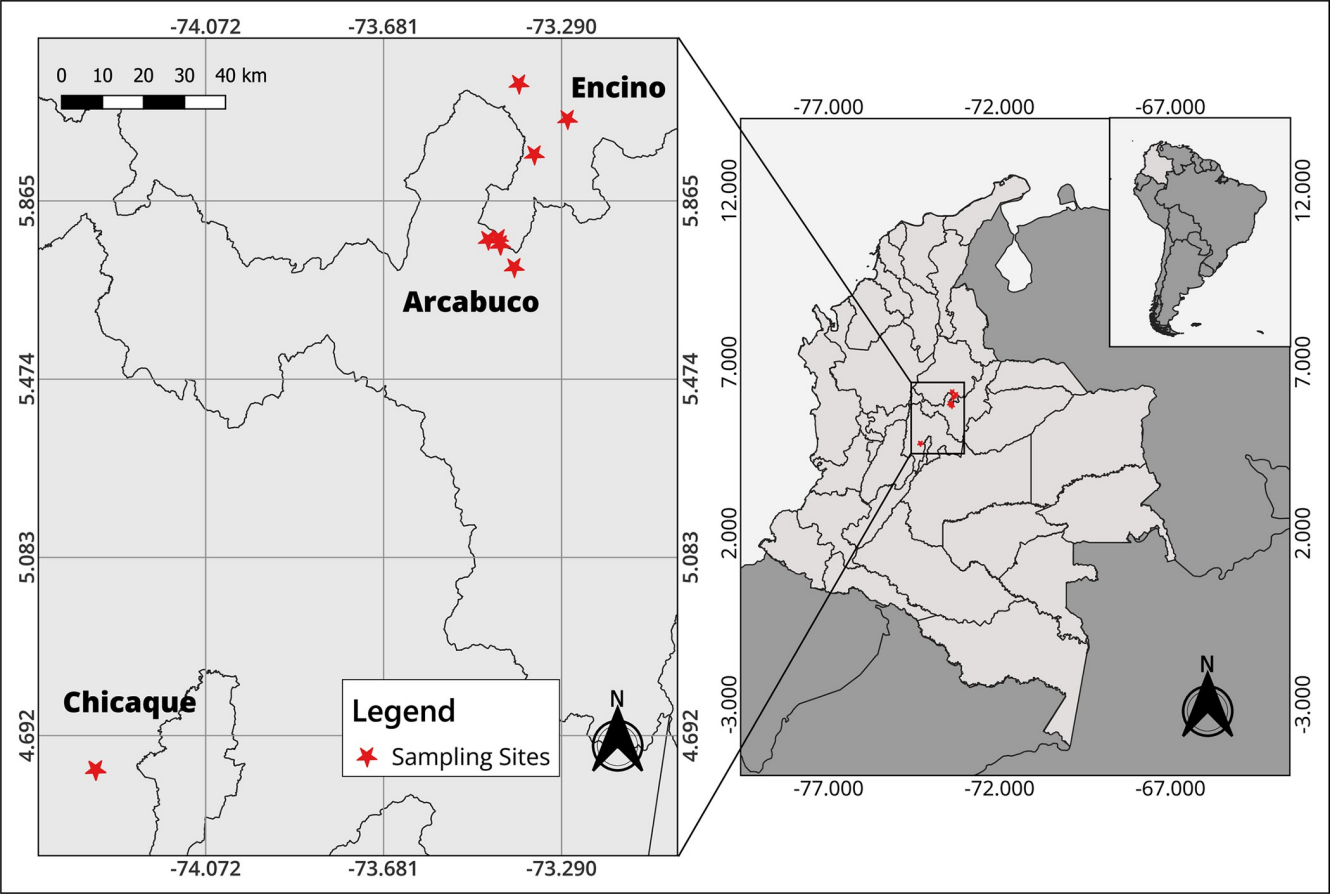
Study site distribution in three different populations of *Q*. *humboldtii* (Arcabuco, Chicaque, and Encino) along the Eastern Andean Cordillera of Colombia. The Colombian and South American layers used were downloaded from [33, 34], respectively.

This study was conducted in three different populations of *Q*. *humboldtii* along a climatic gradient in the Cordillera of the Eastern Andes of Colombia (Fig 1). The first population is situated in the municipality of Arcabuco, in the Madre Monte natural reserve (5°46´9” N, 73°25´28” W). The average temperature of the reserve is 14°C and annual precipitation is between 1,000 and 1,900 mm [30]. Sampling at this site was conducted in an area of approximately 1.5 ha. The second population is located in the Parque Natural Chicaque (4°37’3" N, 74°18’49" W) within the municipality of San Antonio del Tequendama, Department of Cundinamarca. The average temperature at this site is 14.5°C and the annual precipitation is 2,500 mm [31]. The sampling at this site covered an area of approximately 1 ha. The third population, situated in the Department of Santander, is found in the Guanentá Alto Río Fonce Flora and Fauna Sanctuary (SFF GARF), near the municipality of Encino (6°01’09" N, 73°07’08" W). This site has an average annual temperature of 18°C and annual rainfall between 2,500 to 3,000 mm [32]. Sampling at this site was conducted in an approximate area of 1.5 ha. All sampled Andean oak forests were in altitudes between 2,250 and 2,700 m.

### Functional trait sampling and measurement

During the wet season of November 2021 and March 2022, six functional traits related to plant growth and survival were recorded following standardized protocols [35]. A total of 20 adult and 20 juvenile individuals were sampled from each population. The six traits analyzed were leaf area (LA), specific leaf area (SLA), leaf thickness (LT), leaf dry matter content (LDMC), wood density (WD), and specific root length (SRL). A more in depth description of these functional traits and the associated function is in S1 Table.

For leaf traits, three healthy leaves per individual (excluding the petiole) were measured. All leaf measurements were conducted in the same light environment (shade) to maintain consistent conditions for both juveniles and adults. Leaf area (LA, cm^2^) was calculated by scanning fresh leaves and using ImageJ digital image processing software [36]. Specific leaf area (SLA, cm^2^ g^-1^) was then determined by dividing LA by the leaf dry weight. Leaf dry weight was obtained by drying the leaves in an oven at 70°C for 72 h and weighing them immediately after removal from the oven. Leaf thickness (LT, mm) was measured at the sampling site using a digital Vernier caliper, with three measurements averaged for each leaf (always consistent in the different individuals). Wood density (WD, g cm^-3^) was determined by sampling wood from three branches per individual and storing them in silica gel. To calculate WD, the fresh volume of the wood was estimated by immersing the samples in water and rehydrating them for 24 h. The volume was determined using the water displacement method [37]. The wood samples were then dried in an oven at 70°C for 72 h and weighed immediately after removal from the oven [37]. Finally, for specific root length (SRL, cm g^-1^), fine roots from the same individuals were sampled, stored in plastic bags, and refrigerated. The roots were cleaned with tap water and scanned using an Epson Perfection V19 scanner. Subsequently, the root samples were dried at 70°C for 48 h and weighed immediately after removal from the oven. The RhizoVision Explorer software [38] was utilized to estimate SRL.

### Environmental variables

Data analysis was carried out in R v4.0.3 [39]. To better understand the climatic gradient in the study area, we performed a principal component analysis (PCA) using the FactoMineR v2.4 [40] and factoextra v1.0.7 packages [41]. We included seven environmental variables: Max Temperature of Warmest Month (Tmax), Min Temperature of Coldest Month (Tmin), Temperature Seasonality (TS), Precipitation of Wettest Month (Pmax), Precipitation of Driest Month (Pmin), Precipitation Seasonality (PS), and Elevation (Elev). The bioclimatic and elevation data were obtained from WorldClim [42] with a spatial resolution of 30 seconds (~1 km^2^).

### Functional traits analysis

We also created separate PCAs for adult and juvenile functional trait data to visualize the differences in each ontogenetic stage and in each population. To assess the differences between populations (H1), we performed an analysis of variance (ANOVA) for each trait at each ontogenetic stage (adult and juvenile), after checking for normality assumptions and log-transforming the data when necessary (S2 Table). Additionally, we conducted t-tests to compare traits between adults and juveniles within each population (Arcabuco, Chicaque, and Encino; H2). In the few cases where data transformation was not sufficient to meet the assumptions of t-tests, we employed a nonparametric test (S3 Table).

As a post-hoc analysis, we conducted a linear model assessment using the R function "lm" incorporating two categorical factors, ontogeny and population, and their interaction. In handling categorical variables, R employed dummy coding, which generates k-1 binary variables with values of zero and one to represent essential group membership information for a categorical variable with k levels [43]. This allows the categories to serve as distinct predictor variables in the model. The coefficients associated with the dummy variables indicate how each category affects the response variable compared to the reference group (represented as 0 in all dummy variables). In this case, the reference category was set to the juveniles of Arcabuco. The assigned factor values can be found in Table 1.

**Table 1:** Factor’s values assigned in the multiple linear regression models.

Variables	Value
LT	log transformed value
LA	log transformed value
SLA	log transformed value
LDMC	log transformed value
WD	log transformed value
SRL	log transformed value
Juvenile	0 = no, 1 = yes
Chicaque	0 = no, 1 = yes
Encino	0 = no, 1 = yes

The Estimated Regression Eq (1) for all models is as follows:

$$E(Trait)=\beta_{0}+\beta_{1}X_{1}+\beta_{2}X_{2}+\beta_{3}X_{3}+\beta_{4}X_{1}X_{2}+\beta_{5}X_{1}X_{3}$$ (1)

Where β0 represents the mean value of the reference group, X1 represents the value of Juveniles, X2 represents the value of Chicaque, and X3 represents the value of Encino. The coefficients β1, β2, and β3 represent the difference between β0 and the mean value of the groups coded 1 in each reference group. β4 and β5 represent the additive effects of X1 with X2 and X3, respectively.

### External filtering analysis

In order to understand the influence of external filters on the assemblage of *Q*. *humboldtii* populations (H3), we followed the T statistics approach of Violle et al. [29], which is based on the comparison of variances between different organizational levels. In this approach, three statistics are calculated to assess the strength of both internal and external filters on community assembly: T_IP.IC reflects the impact of internal filters by comparing populations variance to total community variance at the intraspecific level; T_IC.IR indicates the influence of external filters by contrasting communities variance with total regional pool variance at the intraspecific level; and T_PC.PR demonstrates the influence of external filters by comparing communities variance to total regional pool variance at the interspecific level. However, given our study’s focus on a single species forming monodominant forests, with more than 80% of the abundance, we excluded the calculation of T_IP.IC and T_PC.PR. Instead, we adapted T_IC.IR to assess the influence of external filters at the intraspecific level on the population assembly, rather than the community assembly (referred to as T_IP.IR). T_IP.IR was determined as the ratio between the population’s variance and the total regional pool’s variance at the individual level. We defined a population as all sampled individuals from a specific location (Arcabuco, Chicaque or Encino), and the regional pool as the aggregate of individuals from all three populations. This approach allows us to evaluate the influence of external filters on *Q*. *humboldtii* individuals for each functional trait at both ontogenetic stages (H3).

To estimate the influence of external filters on Andean oak forest, we further examined whether the different *Q*. *humboldtii* populations are random subsets. To do so, we compared the observed values of T_IP_IR with simulated populations (n = 1000 randomizations) by calculating the standardized effect size (SES), Eq (2).

$$SES=\frac{I_{obs}-I_{sim}}{\sigma_{sim}}$$ (2)

where I_obs_ represents the mean value of the observed values, while I_sim_ and σ_sim_ correspond to the mean value and standard deviation of the random values, respectively. The standardized effect size (SES) quantifies the deviation between observed and simulated populations. When compared to random expectations, negative or positive SES values indicate lower or higher T_IP.IR values, respectively. The significance of the SES was determined using two one-sided tests (lower and upper bound) with a significance level of 5%. This analysis was conducted using the R package cati v.099.4 [44].

## Results

### Environmental gradient

**Fig 2:**
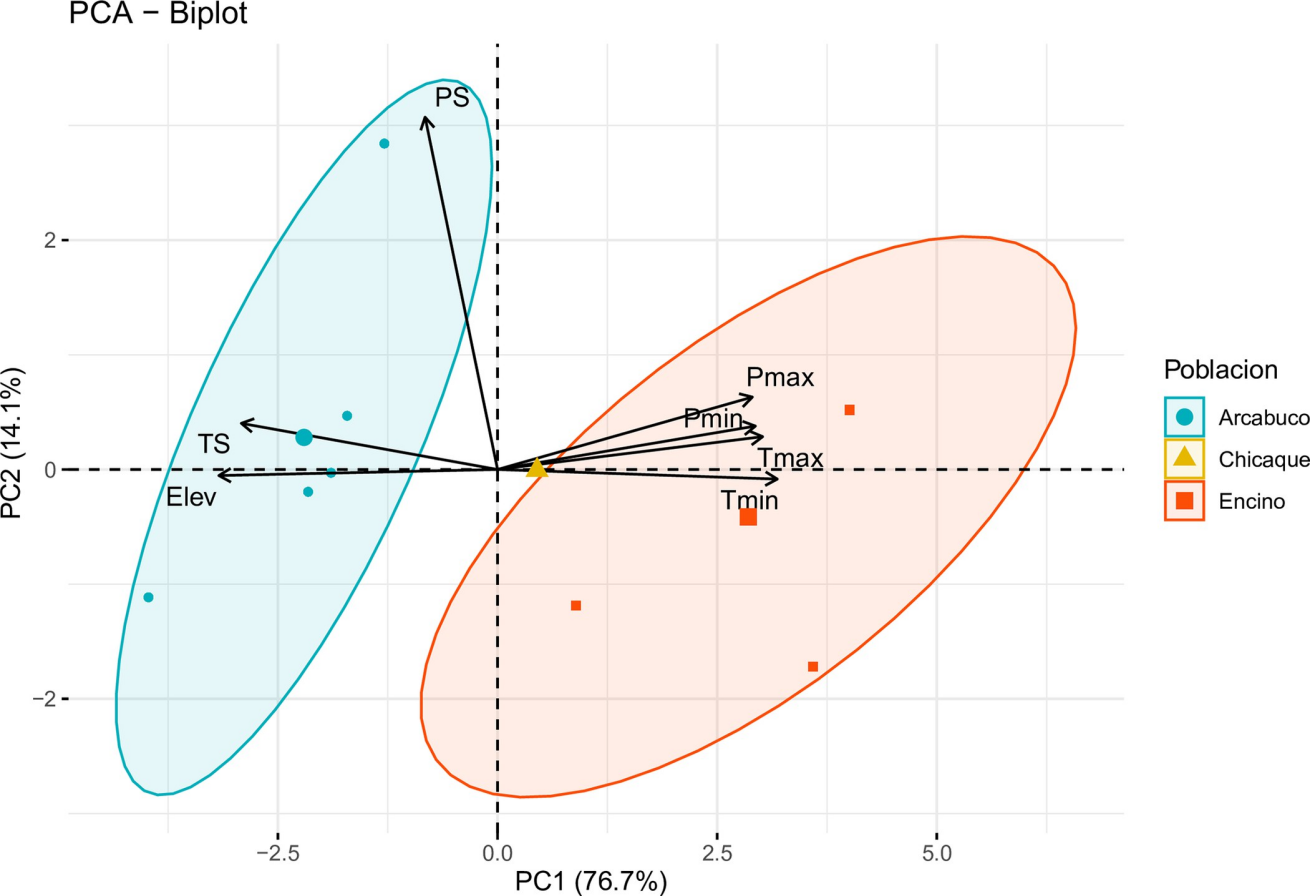
Principal component analysis (PCA) showing the relationships between seven environmental variables and three localities: Arcabuco (represented by blue circles), Chicaque (depicted by yellow triangles), and Encino (shown as orange squares). The environmental variables include Tmax (Max Temperature of Warmest Month), Tmin (Min Temperature of Coldest Month), TS (Temperature Seasonality), Pmax (Precipitation of Wettest Month), Pmin (Precipitation of Driest Month), PS (Precipitation Seasonality), and Elev (Elevation).

The principal component analysis (PCA) of the seven environmental variables yielded a first principal component (PC1) that accounted for 77% of the total variation. PC1 exhibited significant correlations with Tmin, Elev, Tmax, Pmin, TS, and Pmax (Fig 2). Arcabuco, positioned on the left side of the axis, exhibited higher altitudes, pronounced temperature seasonality, and lower temperature and precipitation values. Conversely, Encino was situated on the right side of the axis and Chicaque occupied an intermediate position along the gradient. The second principal component (PC2) explained approximately 14% of the variation and showed a strong correlation with PS (Fig 2). Overall, the ordination analysis illustrated a dominant gradient represented by PC1, reflecting distinct variations in elevation, temperature, and precipitation among the three sites, with Arcabuco and Encino situated at opposite ends of the gradient.

### Functional trait differentiation across populations and ontogeny

**Fig 3:**
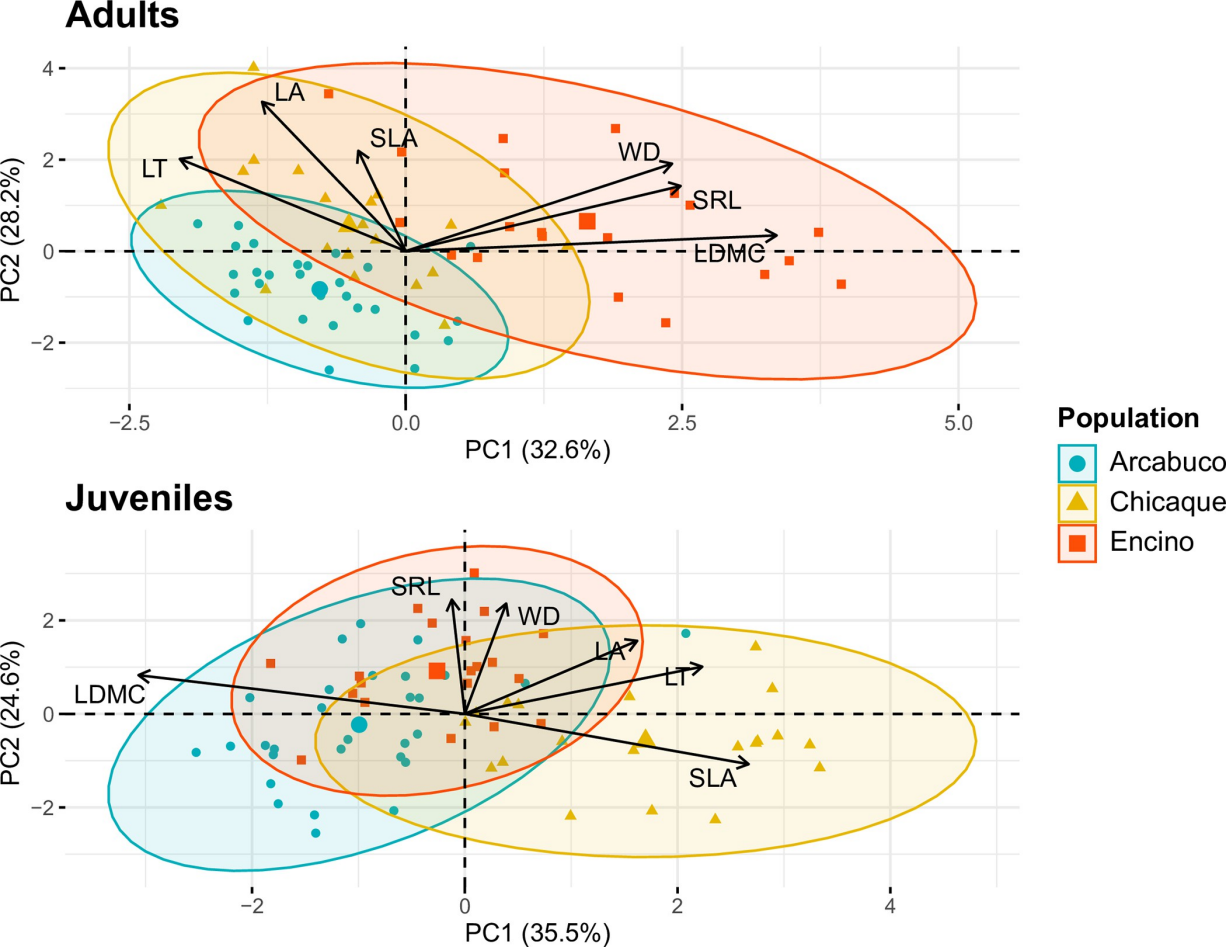
Principal Component Analysis (PCA) illustrating the relationship between the six functional traits values and three distinct populations in adults (upper panel) and juveniles (lower panel) of *Q*. *humboldtii*. Each data point corresponds to an individual sampled from the population, with different colors and shapes representing the respective populations. The functional traits include leaf thickness (LT), leaf area (LA), specific leaf area (SLA), leaf dry matter content (LDMC), wood density (WD), and specific root length (SRL).

PCA analyses for the functional traits of *Q*. *humboldtii* revealed distinct distributions between adults and juveniles (Fig 3). The adult PCA resulted in two principal components (PC1 and PC2) that explain approximately 33% and 28% of the total variation, respectively. PC1 was mainly influenced by LDMC, SRL, and WD, while PC2 exhibited stronger associations with LA and SLA (Fig 3). Although there is some overlap among the functional traits across the three adult populations, there are differences particularly between Encino and the other populations. Encino, positioned on the right side of the axis, displayed higher values for LDMC, SRL, and WD, while Arcabuco and Chicaque have relatively lower values for these traits. Notably, Arcabuco exhibited a more confined trait distribution and overlapped with Chicaque.

In contrast, the PCA analysis of juveniles resulted in PC1 explaining 35.5% of the variation and PC2 explaining 24.6% of the variation. PC1 demonstrated strong associations with LDMC, SLA, and LT, while PC2 showed closer relationships with SRL and WD (Fig 3). Similarly to the adult analysis, the three populations exhibited some overlap in their trait distributions. However, unlike the adult PCA, Chicaque differentiated from the other populations. In PC1, Chicaque had lower values of LDMC and higher values of SLA and LT, while Encino and Arcabuco had higher values of LDMC. Despite the substantial overlap between Encino and Arcabuco, it is noteworthy that Encino displayed a more restricted distribution pattern.

**Fig 4:**
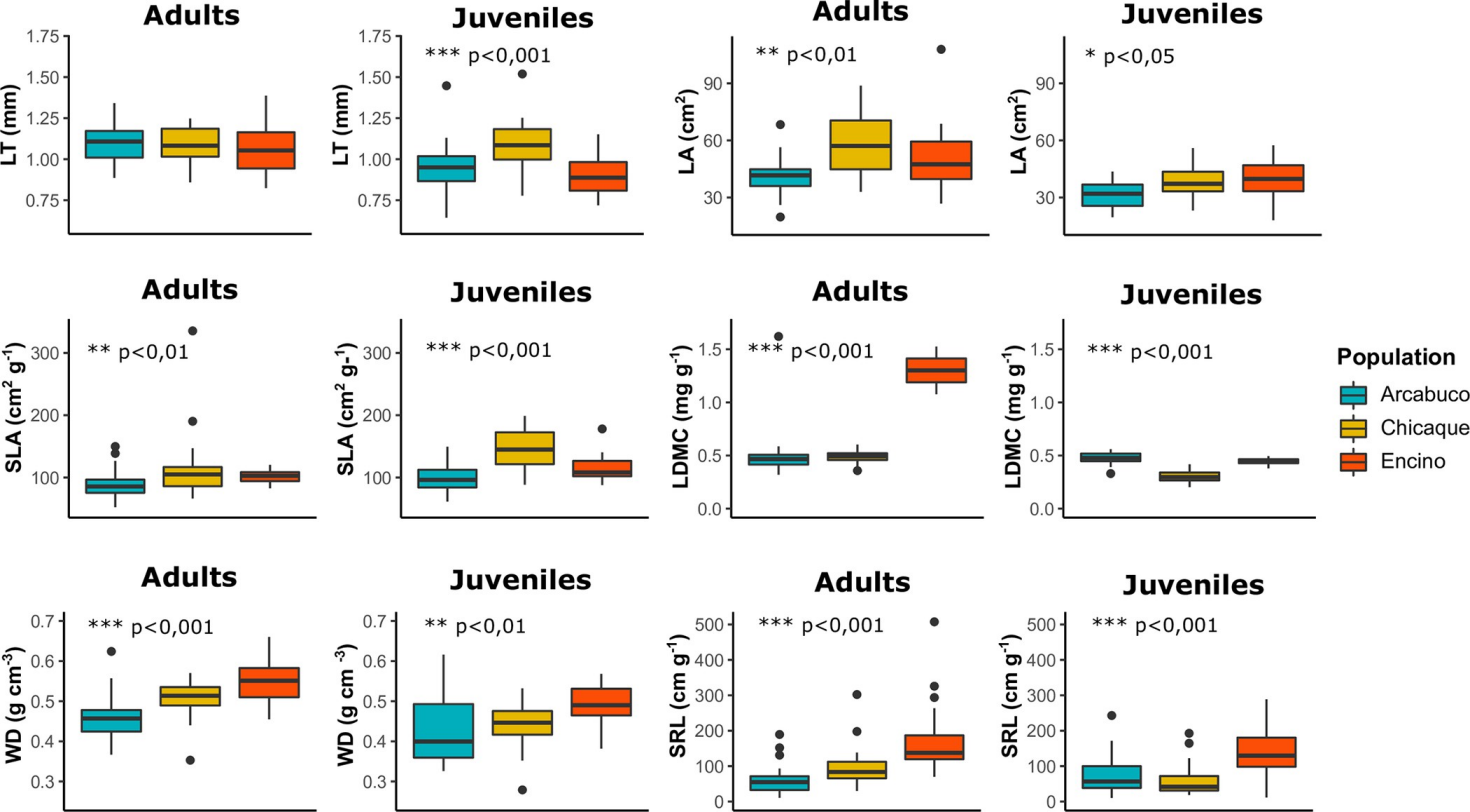
Functional traits within and between populations for adults and juveniles. Significant differences are indicated by asterisks: * denotes p<0.05, ** denotes p<0.01, and *** denotes p<0.001. The functional traits analyzed include leaf thickness (LT), leaf area (LA), specific leaf area (SLA), leaf dry matter content (LDMC), wood density (WD), and specific root length (SRL).

Significant differences were observed in most functional traits when analyzing them individually. Among adult populations (H1), all traits showed significant differences except for leaf thickness (LT) (p>0.05) (Fig 4; S2 Table). Similarly, when comparing juvenile populations, all traits exhibited significant differences (p<0.05) (Fig 4; S2 Table). There were also significant differences (p<0.01) between adults and juveniles within populations, in most of the functional traits (H2). While Chicaque and Encino displayed significant differences in five out of the six analyzed traits, Arcabuco showed significant differences in three traits (S3 Table).

The regression models yielded significant results for all traits (9.21 ≤ F ≤ 12.99, but F = 215.80 for LDMC; p<0.001; S4–S9 Tables), explaining between 26% and 32% of the trait variation in most cases (0.26 ≤ R2 ≤ 0.33; S4–S9 Tables). The model for LDMC demonstrated a high explanatory power, accounting for 89% of the variation (R2 = 0.89; S7 Table). Ontogeny showed a significant effect on four out of the six traits (LT, LA, SLA, and WD), while population exerted a significant effect on all traits, except LT (S4–S9 Tables). The interaction effect between the two factors was significant only for LT, LDMC, and SRL (S4–S9 Tables).

### Influence of external filters on *Quercus humboldtii* populations

**Fig 5:**
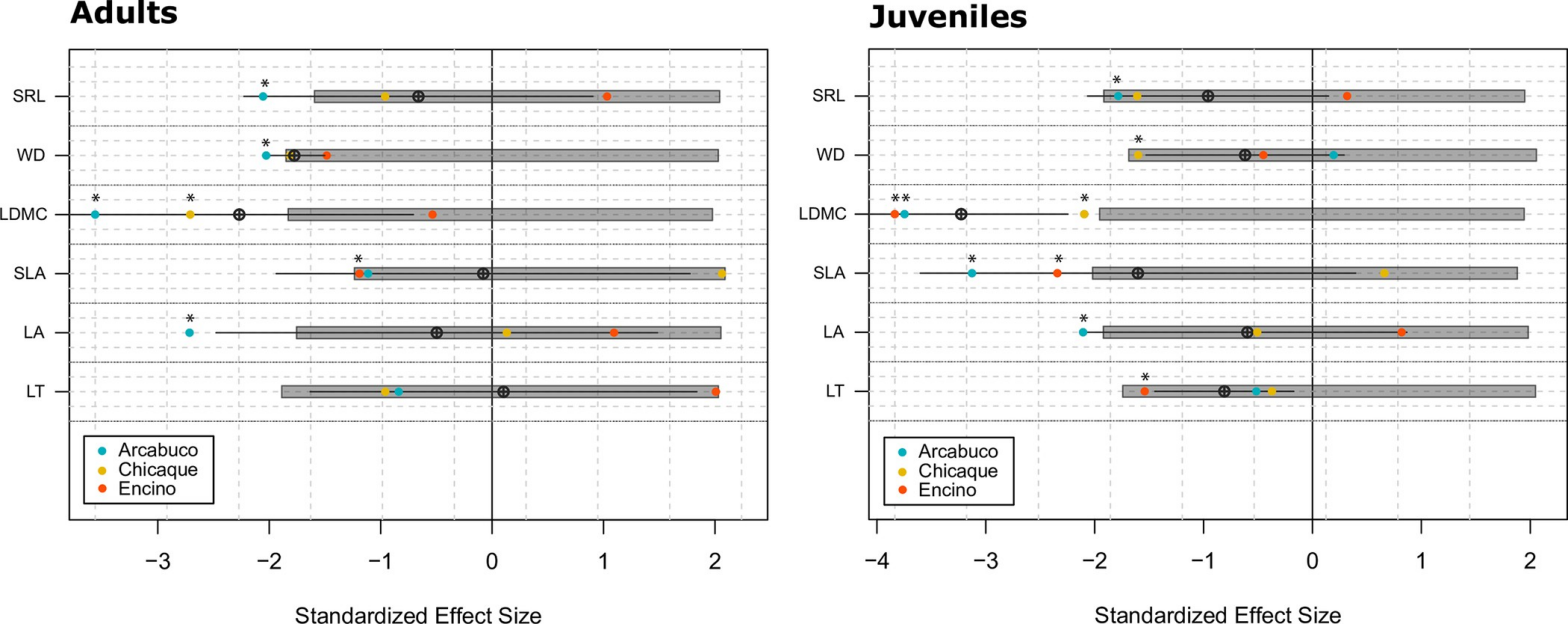
Standardized effect size (SES) of the T_IP.IR for the six functional traits studied. Solid circles represent the SES values for each population (Arcabuco in blue, Chicaque in yellow, and Encino in red). Crossed circles and associated line segments represent the mean and standard deviation of the SES values. The boxes delimit the confidence interval of each null model. If the SES of each population (solid circles) or the mean SES (crossed circles) are significantly different from the random distribution, these differences are denoted with asterisks (see S10 Table for p-values). The functional traits represented include leaf thickness (LT), leaf area (LA), specific leaf area (SLA), leaf dry matter content (LDMC), wood density (WD), and specific root length (SRL). The T_IP.IR statistic is the observed ratio between the population’s variance and the total regional pool variance at the individual level (individuals-populations/regional pool).

The relationship between the SES of T_IP.IR for each functional trait and the simulated null expectation is depicted in Fig 5, with corresponding p-values in S10 Table (H3). In the juvenile stage, significant differences (p<0.05) were observed in four out of the six traits for Arcabuco (LA, SLA, LDMC, and SRL); three for Encino (LT, SLA, LDMC); and two for Chicaque (LDMC, WD). In adults, Chicaque only exhibited significant differences in LDMC, whereas Encino showed significant differences in SLA. Arcabuco, on the other hand, had significant differences in LA, LDMC, WD and SRL. In all cases where significant differences were found, the SES values were negative.

## Discussion

According to our findings, there is a remarkable level of functional differentiation among populations of *Q*. *humboldtii*, consistent with our first hypothesis (H1; Figs 3 and 4; S2 Table). Our analyses also revealed significant differences in multiple traits between adult and juvenile individuals within each population, in line with our second hypothesis (H2; S3 Table). These findings provide strong support for our hypothesis that local differences play a crucial role in driving functional differentiation and facilitating the broad climatic adaptation of *Q*. *humboldtii*. However, it is important to note that local differences alone do not fully account for the observed functional variations in this species. While population differences explained variations in five out of the six analyzed traits, ontogeny accounted for differences in four traits (S4–S9 Tables; S1 Fig). Moreover, consistent with our last hypothesis (H3), we observed a greater impact of external filters on individuals from Arcabuco compared to those from Chicaque and Encino, with a tendency for these filters to have a stronger effect on juveniles than on adults (Fig 5).

### Functional trait variation in Andean oak forests

Considering our results, Andean oak populations in the Colombian Andes differ in several plant functional traits. These traits may provide information to understand the broad climatic adaptation of these species. Previous studies on functional traits of *Q*. *humboldtii* have also shown that traits, such as SLA and WD, separate between sites as well, especially in an elevational gradient [18].

Based on the overall trait patterns [19, 20], we expected that individuals from populations with more extreme conditions would exhibit traits indicative of a more conservative life strategy (H1). This was the case for Arcabuco, which is characterized by a more pronounced temperature and precipitation seasonality (Fig 2), compared to Chicaque and Encino. Our findings supported this hypothesis, particularly for juveniles. In the juvenile stage, traits such as LA, SLA, and SRL showed significantly lower values in Arcabuco compared to Chicaque or Encino, while LDMC values were higher (Figs 3 and 4). These trait combinations suggest a resource conservation strategy that could confer advantages in resource-limited or challenging environments [19].

The observed reductions in LA and SLA, along with the corresponding increase in LDMC, enable Arcabuco juveniles to minimize water loss through transpiration and provide protection against low temperatures and wind by having smaller-sized leaves with a higher concentration of dry matter [23, 24]. Furthermore, the lower SRL values observed in Arcabuco indicate the presence of longer and thicker roots, facilitating an extensive exploration of the soil to extract scarce water resources [22] and supporting ectomycorrhizal symbiosis [45, 46]. By contrast, in the populations of Chicaque and Encino, high values of LA and SLA, sometimes coupled with low LDMC or high SRL (Fig 4), suggest strategic adaptations aimed at optimizing resource acquisition, promoting rapid growth, and facilitating efficient nutrient turnover [19, 22]. This is beneficial in environments characterized by abundant resources and favorable climatic conditions.

In adults, individuals from Arcabuco exhibit significantly lower LDMC and WD values compared to the other two populations, indicating a more acquisitive strategy (Fig 4; S2 Table). This finding is unexpected, as it is typically associated with plants thriving in resource-rich environments [19]. The contrasting trend in the adults could be attributed to nutrient availability influenced by mycorrhizal associations [46]. In Arcabuco, mycorrhizal associations may enable juveniles and adults to maintain a similar functional strategy, with relatively stable LDMC and WD traits between the two stages. In contrast, Chicaque and Encino show a transition from efficient resource acquisition in juveniles to a conservative strategy emphasizing resource conservation and long-term persistence in adults (S3 Table). The occurrence of ontogenetic shifts in ecological strategies has been documented in previous studies [47], and support the results obtained in this study (H2). These shifts are usually associated with higher growth rates in juveniles, which in turn increase the probability of individuals reaching a functionally specialized stage that is more resilient to disturbances [48]. Other studies have also emphasized the significance of environmental factors and ontogeny in shaping functional traits at the intraspecific level [47, 49].

### Influence of external filtering on population assembly in Andean oak forest

The analysis of external filters’ influence on Andean oak forests revealed a stronger effect in Arcabuco compared to the other populations (Fig 5), supporting our hypothesis that populations facing higher environmental stress would undergo greater external filtering. Chicaque and Encino populations, benefiting from more favorable climatic conditions, exhibited a diminished impact of external filters, resulting in a more stochastic distribution of traits (Fig 5). This pattern was particularly evident among adult individuals, as both Arcabuco and Encino juveniles experienced stronger external filtering compared to Chicaque (Fig 5). Contrary to previous studies [46, 48], we found a stronger influence of environmental filtering on juveniles rather than adults, particularly in traits such as LDMC and SLA (Fig 5).

These findings suggest that external filters are differentially influencing population assembly processes in Andean oak forests, ultimately resulting in distinct trait distributions among populations and ontogenetic stages (Figs 3 and 4). In Chicaque and Encino, where external filtering is less influential and allows for greater functional differentiation between individuals, we propose that the storage effect plays a pivotal role. The ontogeny-dependent functional transition within these populations may give rise to temporal niche differentiations, offering several advantages [27, 50]. First, it would decrease intraspecific competition by creating favorable conditions for efficient resource access and utilization, resulting in enhanced survival, growth, and reproduction. Second, it would contribute to population stability by buffering *Q*. *humboldtii* against resource fluctuations, ensuring its persistence even during periods of scarcity. This stability would foster the long-term success and dominance of *Q*. *humboldtii* within the community. Lastly, it would promote the coexistence of *Q*. *humboldtii* with other species by creating niches and opportunities for diverse resource requirements, thereby fostering a resilient and diverse ecosystem.

Conversely, in Arcabuco, where external filtering exerts a stronger influence and constrains trait variability, population assembly dynamics may be influenced by facilitation and other associated processes, thereby enabling the maintenance of a functional strategy in adults that resembles the one observed in juveniles (S3 Table). Research has shown that plants with higher LDMC and lower SLA and SRL values (i.e., Arcabuco) tend to exhibit higher rates of colonization by ectomycorrhizal fungi (ECM) [46]. This symbiotic association with mycorrhizal fungi provides a significant advantage to plants in resource-limited environments by expanding the absorption area of plant roots through external fungal mycelium, enhancing water and nutrient uptake. In contrast, plants with high SLA and SRL values (i.e., Chicaque and Encino) are likely adapted to resource-abundant environments, where they can acquire resources directly at a lower cost, without relying heavily on fungal symbiosis [46]. Although the role of facilitation in the studied communities remains to be evaluated, the importance of *Q*. *humboldtii* as an ECM reservoir has already been emphasized [51].

### Evolutionary history can explain intraspecific variation

The population differences between *Q*. *humboldtii* raise questions regarding their connectivity and the genetic structure of its populations. Hooghiemstra and Flantua [52] reported that the upper limit of the forest reached an altitude of 2,000 m during the last glacial maxima (21 ka BP to ca. 14 ka BP). The upper mountain forest was compressed by about 400 m and displaced to lower elevations compared to the current altitudes. The lower mountain forest was also displaced altitudinally to lower altitudes. The demography and genetics of *Q*. *humboldtii* in the Andean montane forests were affected in various ways by these variations. In particular, palynological and floristic investigations have suggested migration routes that allowed the colonization of different mountain ranges [12]. This would imply, that the Andean oak forests may have remained connected over time and that populations of *Q*. *humboldtii* would have maintained gene flow [9].

A recent study by Zorrilla-Azcué et al. [9], demonstrates that this species has a lack of genetic structure and considerable genetic diversity. They found eight genetic clusters distributed along the three cordilleras (Western, Central, and Eastern). Two of their study sites correspond to sampled sites in this study (Encino and Arcabuco) and both are in two different genetic clusters (Fig 1). These findings show that although the Andean oak may be acclimating to their environment, there could also be differences responding to their genetic diversity and evolutionary history (and thus, adaptation). Evolutionary history, in addition to environmental factors, can be affecting trait values and could explain the functional trait differences between the three populations studied (Figs 3–5). Within the *Quercus* genus, the influence of the environment has been proposed to surpass that of genetic relationships [53]. The question remains as to how the functional traits of these populations will change in the future, as fragmentation increases and there are more restrictions to gene flow.

## Conclusions

*Quercus humboldtii* displays significant variation in functional traits among populations in the Eastern cordillera of the Colombian Andes. The wide intraspecific trait variation observed in this species likely contributes to its adaptability to diverse climates and environments. These results underscore the intricate interplay between environmental conditions, ontogenetic stage, and trait variation, highlighting the dynamic and context-dependent nature of functional traits within Andean oak forests. Additionally, the observed functional differences may be influenced by the species’ evolutionary history and genetic differentiation among populations. Thus, it is crucial to consider environmental, evolutionary, and developmental factors when investigating intraspecific variation in functional traits. By integrating these factors into future research approaches, a more comprehensive understanding of the dynamic interactions between organisms and their environment can be achieved, enhancing our knowledge of ecological patterns and processes.

## Supporting information

S1 Fig Predictor effect plot showing the influence of two predictor variables (population and ontogeny) on six functional traits. Asterisks indicate a significant effect of the independent variables included in the multiple linear models: ontogeny (o), population (p), and their interaction (i). Significant differences are indicated by asterisks: ** denotes p<0.01 and ***denotes p<0.001. LT = leaf thickness; LA = leaf area; SLA = specific leaf area; LDMC = leaf dry matter content; WD = wood density; SRL = specific root length.
(DOCX)

S1 Table General description of the functional traits included in this study, which details the acronyms, units of measurement and the associated function. Trait information is based on Pérez-Harguindeguy et al. 2013 and Garnier et al. 2015.
(DOCX)

S2 Table Summary table of the population values and tests for adults and juveniles. It includes mean values and standard deviations, p-values of the tests used to assess normality (Shapiro-wilk and Levene’s test), as well as the p-values of the ANOVAs. When we obtained p<0.05 in Levene’s tests, we performed Welch’s ANOVA. LT = leaf thickness; LA = leaf area; SLA = specific leaf area; LDMC = leaf dry matter content; WD = wood density; SRL = specific root length. Significant differences are indicated by asterisks: * denotes p<0.05, ** denotes p<0.01, and *** denotes p<0.001.
(DOCX)

S3 Table Summary table of adult and juvenile values and tests for each population. In includes mean values and standard deviations, p-values of the tests used to assess normality (Shapiro-wilk and Levene’s test), as well as p-values of the t-test or Mann Whitney U-test. When we obtained p<0.05 in the Levene’s tests, we performed a Welch’s t-test. LT = leaf thickness; LA = leaf area; SLA = specific leaf area; LDMC = leaf dry matter content; WD = wood density; SRL = specific root length. Significant differences are indicated by asterisks: * denotes p<0.05, ** denotes p<0.01, and *** denotes p<0.001.
(DOCX)

S4 Table Effect of ontogeny, population, and their interaction on leaf thickness (LT). As the two independent variables are categorical, they were dummy coded, and the reference group was set to the adults of Arcabuco. The coefficients (β) represent the change in the dependent variable (the trait) for each category of the dummy variable, compared to the reference category. The standard errors (SE) quantify the variability or uncertainty of the coefficient estimates. The t-values (t) assess the significance of each coefficient estimate, while the associated p-values (P) determine their statistical significance. * denotes p<0.05, ** denotes p<0.01, and *** denotes p<0.001. The constant represents the value of the dependent variable when all predictor variables are at their reference level. The R-squared (R2) value indicates the proportion of variance explained by the predictors, while the F-statistic and its associated p-value test the overall significance of the regression model.
(DOCX)

S5 Table Effect of ontogeny, population, and their interaction on leaf area (LA). As the two independent variables are categorical, they were dummy coded, and the reference group was set to the adults of Arcabuco. The coefficients (β) represent the change in the dependent variable (the outcome) for each category of the dummy variable, compared to the reference category. The standard errors (SE) quantify the variability or uncertainty of the coefficient estimates. The t-values (t) assess the significance of each coefficient estimate, while the associated p-values (P) determine their statistical significance. * denotes p<0.05, ** denotes p<0.01, and *** denotes p<0.001. The constant represents the value of the dependent variable when all predictor variables are at their reference level. The R-squared (R2) value indicates the proportion of variance explained by the predictors, while the F-statistic and its associated p-value test the overall significance of the regression model.
(DOCX)

S6 Table Effect of ontogeny, population, and their interaction on specific leaf area (SLA). As the two independent variables are categorical, they were dummy coded, and the reference group was set to the adults of Arcabuco. The coefficients (β) represent the change in the dependent variable (the outcome) for each category of the dummy variable, compared to the reference category. The standard errors (SE) quantify the variability or uncertainty of the coefficient estimates. The t-values (t) assess the significance of each coefficient estimate, while the associated p-values (P) determine their statistical significance. * denotes p<0.05, ** denotes p<0.01, and *** denotes p<0.001. The constant represents the value of the dependent variable when all predictor variables are at their reference level. The R-squared (R2) value indicates the proportion of variance explained by the predictors, while the F-statistic and its associated p-value test the overall significance of the regression model.
(DOCX)

S7 Table Effect of ontogeny, population, and their interaction on leaf dry matter content (LDMC). As the two independent variables are categorical, they were dummy coded, and the reference group was set to the adults of Arcabuco. The coefficients (β) represent the change in the dependent variable (the outcome) for each category of the dummy variable, compared to the reference category. The standard errors (SE) quantify the variability or uncertainty of the coefficient estimates. The t-values (t) assess the significance of each coefficient estimate, while the associated p-values (P) determine their statistical significance. * denotes p<0.05, ** denotes p<0.01, and *** denotes p<0.001. The constant represents the value of the dependent variable when all predictor variables are at their reference level. The R-squared (R2) value indicates the proportion of variance explained by the predictors, while the F-statistic and its associated p-value test the overall significance of the regression model.
(DOCX)

S8 Table Effect of ontogeny, population, and their interaction on wood density (WD). As the two independent variables are categorical, they were dummy coded, and the reference group was set to the adults of Arcabuco. The coefficients (β) represent the change in the dependent variable (the outcome) for each category of the dummy variable, compared to the reference category. The standard errors (SE) quantify the variability or uncertainty of the coefficient estimates. The t-values (t) assess the significance of each coefficient estimate, while the associated p-values (P) determine their statistical significance. * denotes p<0.05, ** denotes p<0.01, and *** denotes p<0.001. The constant represents the value of the dependent variable when all predictor variables are at their reference level. The R-squared (R2) value indicates the proportion of variance explained by the predictors, while the F-statistic and its associated p-value test the overall significance of the regression model.
(DOCX)

S9 Table Effect of ontogeny, population, and their interaction on specific root length (SRL). As the two independent variables are categorical, they were dummy coded, and the reference group was set to the adults of Arcabuco. The coefficients (β) represent the change in the dependent variable (the outcome) for each category of the dummy variable, compared to the reference category. The standard errors (SE) quantify the variability or uncertainty of the coefficient estimates. The t-values (t) assess the significance of each coefficient estimate, while the associated p-values (P) determine their statistical significance. * denotes p<0.05, ** denotes p<0.01, and *** denotes p<0.001. The constant represents the value of the dependent variable when all predictor variables are at their reference level. The R-squared (R2) value indicates the proportion of variance explained by the predictors, while the F-statistic and its associated p-value test the overall significance of the regression model.
(DOCX)

S10 Table P-values of the two one-sided statistical tests performed (lower and upper limit) to compare T_IP.IR from each population with random expectations. LT = leaf thickness; LA = leaf area; SLA = specific leaf area; LDMC = leaf dry matter content; WD = wood density; SRL = specific root length. Bold indicates significant differences (p < 0.05).
(DOCX)
