# Supplementary material for: Functional differences between Andean oak (Quercus humboldtii Bonpl.) populations: The importance of intraspecific variation
Source: PLoS One. 2024 Mar 13;19(3):e0299645. doi: 10.1371/journal.pone.0299645 (PMC10936772; doi:10.1371/journal.pone.0299645)
Supplement: S1 Fig — Asterisks indicate a significant effect of the independent variables included in the multiple linear models: ontogeny (o), population (p), and their interaction (i). Significant differences are indicated by asterisks: ** denotes p<0.01 and ***denotes p<0.001. LT = leaf thickness; LA = leaf area; SLA = specific leaf area; LDMC = leaf dry matter content; WD = wood density; SRL = specific root length. (DOCX) [file pone.0299645.s001.docx]

**Supplementary material**


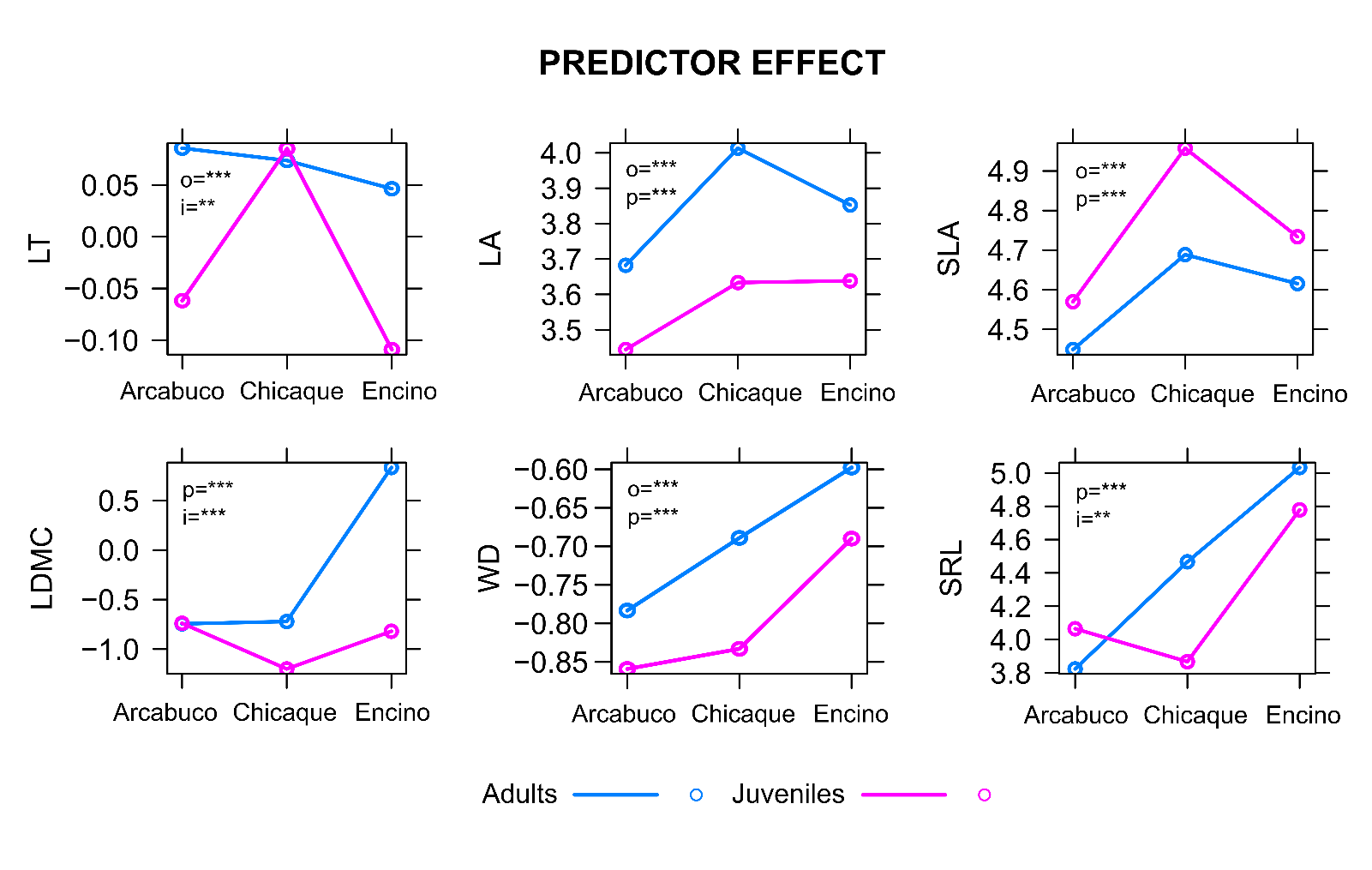


**S1 Fig.** **Predictor effect plot showing the influence of two predictor variables (population and ontogeny) on six functional traits.** Asterisks indicate a significant effect of the independent variables included in the multiple linear models: ontogeny (o), population (p), and their interaction (i). Significant differences are indicated by asterisks: ** denotes p<0.01 and ***denotes p<0.001. LT = leaf thickness; LA = leaf area; SLA = specific leaf area; LDMC = leaf dry matter content; WD = wood density; SRL = specific root length.
