# Supplementary material for: Functional differences between Andean oak (Quercus humboldtii Bonpl.) populations: The importance of intraspecific variation
Source: PLoS One. 2024 Mar 13;19(3):e0299645. doi: 10.1371/journal.pone.0299645 (PMC10936772; doi:10.1371/journal.pone.0299645)
Supplement: S1 Table — Trait information is based on Pérez-Harguindeguy et al. 2013 and Garnier et al. 2015. (DOCX) [file pone.0299645.s002.docx]

**Supplementary material**

**S1 Table.** **General description of the functional traits included in this study, which details the acronyms, units of measurement and the associated function.** Trait information is based on Pérez-Harguindeguy et al. 2013 and Garnier et al. 2015.

| **Functional trait** | **Associated function** |
| --- | --- |
| Leaf area, LA (mm^2^) | Light interception area, respiration, transpiration, gas exchange. |
| Specific leaf area, SLA (mm^2^ mg^-1^) | Relative growth rate, leaf life, leaf nitrogen concentration and photosynthetic capacity. |
| Leaf thickness, Lth (mm) | Physical strength of the leaves, photosynthetic rate and key component of SLA. |
| Leaf dry matter content, LDMC (mg g^-1^) | Construction costs, nutrient retention, resistance against herbivory and physical damage. |
| Wood density, WD (g.cm^-3^) | Construction costs, growth rate, architecture, resistance to pathogens and mortality rate. |
| Specific root length, SRL (cm.g^-1^) | This ratio provides a unit of acquisition (root length) for a dry-mass investment. It is considered as a below-ground analog to SLA and plants with high SRL are considered to have higher rates of nutrient and water uptake. |

References:

Garnier E, Navas M-L, Grigulis K. Plant Functional Diversity: Organism traits, community structure, and ecosystem properties. London, England: Oxford University Press; 2015.

Pérez-Harguindeguy N, Díaz S, Garnier E, Lavorel S, Poorter H, Jaureguiberry P, et al. New handbook for standardised measurement of plant functional traits worldwide. Aust J Bot. 2013;61(3):167. doi: 10.1071/bt12225.
