# Supplementary material for: Functional differences between Andean oak (Quercus humboldtii Bonpl.) populations: The importance of intraspecific variation
Source: PLoS One. 2024 Mar 13;19(3):e0299645. doi: 10.1371/journal.pone.0299645 (PMC10936772; doi:10.1371/journal.pone.0299645)
Supplement: S2 Table — It includes mean values and standard deviations, p-values of the tests used to assess normality (Shapiro-wilk and Levene’s test), as well as the p-values of the ANOVAs. When we obtained p<0.05 in Levene’s tests, we performed Welch’s ANOVA. LT = leaf thickness; LA = leaf area; SLA = specific leaf area; LDMC = leaf dry matter content; WD = wood density; SRL = specific root length. Significant differences are indicated by asterisks: * denotes p<0.05, ** denotes p<0.01, and *** denotes p<0.001. (DOCX) [file pone.0299645.s003.docx]

**Supplementary material**

**S2 Table.** **Summary table of the population values and tests for adults and juveniles.** It includes mean values and standard deviations, p-values of the tests used to assess normality (Shapiro-wilk and Levene's test), as well as the p-values of the ANOVAs. When we obtained p<0.05 in Levene's tests, we performed Welch's ANOVA. LT = leaf thickness; LA = leaf area; SLA = specific leaf area; LDMC = leaf dry matter content; WD = wood density; SRL = specific root length. Significant differences are indicated by asterisks: * denotes p<0.05, ** denotes p<0.01, and *** denotes p<0.001.

| Ontogeny | Trait | n | Mean+SD | | | Shapiro-wilk *p-value* | | | Levene test *p-value* | ANOVA | |
| --- | --- | --- | --- | --- | --- | --- | --- | --- | --- | --- | --- |
|  |  |  | **Arcabuco** | **Chicaque** | **Encino** | **Arcabuco** | **Chicaque** | **Encino** |  | **F** | ***P-value*** |
| Adults | LT | 69 | 0.09±0.11 | 0.07±0.11 | 0.05±0.14 | 0.727 | 0.203 | 0.767 | 0.379 | 0.65 | 0.523 |
|  | LA | 69 | 3.68±0.25 | 4.01±0.29 | 3.85±0.35 | 0.610 | 0.547 | 0.536 | 0.274 | 7.67 | 0.001** |
|  | SLA | 69 | 4.45±0.23 | 4.69±0.35 | 4.62±0.11 | 0.539 | 0.004** | 0.283 | 0.047* | 6.44 | 0.004** |
|  | LDMC | 69 | -0.74±0.28 | -0.72±0.14 | 0.83±0.35 | 0.000*** | 0.207 | 0.942 | 0.040* | 177.17 | 0.000*** |
|  | WD | 69 | -0.78±0.11 | -0.69±0.11 | -0.60±0.09 | 0.366 | 0.003** | 0.967 | 0.915 | 18.68 | 0.000*** |
|  | SRL | 69 | 3.82±0.76 | 4.47±0.52 | 5.03±0.49 | 0.327 | 0.926 | 0.315 | 0.137 | 22.49 | 0.000*** |
| Juveniles | LT | 69 | -0.06±0.16 | 0.09±0.14 | -0.11±0.13 | 0.615 | 0.598 | 0.867 | 0.848 | 9.96 | 0.000*** |
|  | LA | 69 | 3.44±0.25 | 3.63±0.23 | 3.64±0.29 | 0.045* | 0.788 | 0.140 | 0.797 | 4.74 | 0.012* |
|  | SLA | 69 | 4.57±0.21 | 4.96±0.25 | 4.73±0.17 | 0.874 | 0.335 | 0.214 | 0.176 | 20.52 | 0.000*** |
|  | LDMC | 69 | -0.74±0.12 | -1.20±0.21 | -0.82±0.07 | 0.029* | 0.574 | 0.579 | 0.000*** | 39.15 | 0.000*** |
|  | WD | 69 | -0.86±0.19 | -0.83±0.15 | -0.69±0.14 | 0.054 | 0.022* | 0.252 | 0.104 | 6.61 | 0.002** |
|  | SRL | 69 | 4.07±0.70 | 3.87±0.68 | 4.78±0.70 | 0.970 | 0.334 | 0.003 | 0.755 | 9.78 | 0.000*** |
