# Supplementary material for: Functional differences between Andean oak (Quercus humboldtii Bonpl.) populations: The importance of intraspecific variation
Source: PLoS One. 2024 Mar 13;19(3):e0299645. doi: 10.1371/journal.pone.0299645 (PMC10936772; doi:10.1371/journal.pone.0299645)
Supplement: S3 Table — In includes mean values and standard deviations, p-values of the tests used to assess normality (Shapiro-wilk and Levene’s test), as well as p-values of the t-test or Mann Whitney U-test. When we obtained p<0.05 in the Levene’s tests, we performed a Welch’s t-test. LT = leaf thickness; LA = leaf area; SLA = specific leaf area; LDMC = leaf dry matter content; WD = wood density; SRL = specific root length. Significant differences are indicated by asterisks: * denotes p<0.05, ** denotes p<0.01, and *** denotes p<0.001. (DOCX) [file pone.0299645.s004.docx]

**Supplementary material**

**S3 Table.** **Summary table of adult and juvenile values and tests for each population.** In includes mean values and standard deviations, p-values of the tests used to assess normality (Shapiro-wilk and Levene's test), as well as p-values of the t-test or Mann Whitney U-test. When we obtained p<0.05 in the Levene's tests, we performed a Welch's t-test. LT = leaf thickness; LA = leaf area; SLA = specific leaf area; LDMC = leaf dry matter content; WD = wood density; SRL = specific root length. Significant differences are indicated by asterisks: * denotes p<0.05, ** denotes p<0.01, and *** denotes p<0.001.

| Population | Trait | Mean+SD | | shapiro-wilk *p-value* | | Levene test *p-value* | T/W | *p-value* | t-student(T) /Wilcoxon(W) |
| --- | --- | --- | --- | --- | --- | --- | --- | --- | --- |
|  |  | **Adults** | **Juveniles** | **Adults** | **Juveniles** |  |  |  |  |
| Acabuco | LT | 0.09±0.11 | -0.06±0.16 | 0.727 | 0.615 | 0.210 | 4.13 | 0.000*** | T |
|  | LA | 3.68±0.25 | 3.44±0.25 | 0.610 | 0.045* | 0.613 | 3.65 | 0.001*** | T |
|  | SLA | 4.45±0.23 | 4.57±0.21 | 0.539 | 0.874 | 0.960 | -2.09 | 0.041* | T |
|  | LDMC | -0.74±0.28 | -0.74±0.12 | 0.000*** | 0.029* | 0.117 | 361.50 | 0.363 | W |
|  | WD | -0.78±0.11 | -0.86±0.19 | 0.366 | 0.054 | 0.002** | 1.87 | 0.069 | T |
|  | SRL | 3.82±0.76 | 4.07±0.70 | 0.327 | 0.970 | 0.817 | -1.26 | 0.214 | T |
| Chicaque | LT | 0.07±0.11 | 0.09±0.14 | 0.203 | 0.598 | 0.471 | -0.29 | 0.771 | T |
|  | LA | 4.01±0.29 | 3.63±0.23 | 0.547 | 0.788 | 0.255 | 4.56 | 0.000*** | T |
|  | SLA | 4.69±0.35 | 4.96±0.25 | 0.004** | 0.335 | 0.714 | -2.79 | 0.008** | T |
|  | LDMC | -0.72±0.14 | -1.20±0.21 | 0.207 | 0.574 | 0.074 | 8.57 | 0.000*** | T |
|  | WD | -0.69±0.11 | -0.83±0.15 | 0.003** | 0.022* | 0.277 | 337.00 | 0.000*** | W |
|  | SRL | 4.47±0.52 | 3.87±0.68 | 0.926 | 0.334 | 0.207 | 3.13 | 0.003** | T |
| Encino | LT | 0.05±0.14 | -0.11±0.13 | 0.767 | 0.867 | 0.762 | 3.62 | 0.001*** | T |
|  | LA | 3.85±0.35 | 3.64±0.29 | 0.536 | 0.140 | 0.508 | 2.12 | 0.040* | T |
|  | SLA | 4.62±0.11 | 4.73±0.17 | 0.283 | 0.214 | 0.124 | -2.66 | 0.011* | T |
|  | LDMC | 0.83±0.35 | -0.82±0.07 | 0.942 | 0.579 | 0.000*** | 20.84 | 0.000*** | T |
|  | WD | -0.60±0.09 | -0.69±0.14 | 0.967 | 0.252 | 0.237 | 2.40 | 0.021* | T |
|  | SRL | 5.03±0.49 | 4.78±0.70 | 0.315 | 0.003** | 0.479 | 1.33 | 0.192 | T |
