# Supplementary material for: Functional differences between Andean oak (Quercus humboldtii Bonpl.) populations: The importance of intraspecific variation
Source: PLoS One. 2024 Mar 13;19(3):e0299645. doi: 10.1371/journal.pone.0299645 (PMC10936772; doi:10.1371/journal.pone.0299645)
Supplement: S6 Table — As the two independent variables are categorical, they were dummy coded, and the reference group was set to the adults of Arcabuco. The coefficients (β) represent the change in the dependent variable (the outcome) for each category of the dummy variable, compared to the reference category. The standard errors (SE) quantify the variability or uncertainty of the coefficient estimates. The t-values (t) assess the significance of each coefficient estimate, while the associated p-values (P) determine their statistical significance. * denotes p<0.05, ** denotes p<0.01, and *** denotes p<0.001. The constant represents the value of the dependent variable when all predictor variables are at their reference level. The R-squared (R2) value indicates the proportion of variance explained by the predictors, while the F-statistic and its associated p-value test the overall significance of the regression model. (DOCX) [file pone.0299645.s007.docx]

**Supplementary material**

**S6 Table.** **Effect of ontogeny, population, and their interaction on specific leaf area (SLA).** As the two independent variables are categorical, they were dummy coded, and the reference group was set to the adults of Arcabuco. The coefficients (β) represent the change in the dependent variable (the outcome) for each category of the dummy variable, compared to the reference category. The standard errors (SE) quantify the variability or uncertainty of the coefficient estimates. The t-values (t) assess the significance of each coefficient estimate, while the associated p-values (P) determine their statistical significance. * denotes p<0.05, ** denotes p<0.01, and *** denotes p<0.001. The constant represents the value of the dependent variable when all predictor variables are at their reference level. The R-squared (R2) value indicates the proportion of variance explained by the predictors, while the F-statistic and its associated p-value test the overall significance of the regression model.

| **Independent variables** | **β** | **SE** | **t** | **p** |  |
| --- | --- | --- | --- | --- | --- |
| Juvenile | 0.121 | 0.061 | 1.996 | 0.048 | * |
| Chicaque | 0.240 | 0.067 | 3.581 | 0.000 | *** |
| Encino | 0.167 | 0.067 | 2.487 | 0.014 | * |
| Juvenile:Chicaque | 0.148 | 0.095 | 1.566 | 0.120 |  |
| Juvenile:Encino | -0.002 | 0.095 | -0.024 | 0.981 |  |
|  |  |  |  |  |  |
| Constant | 4.449 |  |  |  |  |
|  |  |  |  |  |  |
| R2 | 0.330 |  |  |  |  |
| F-statistic | 12.990 | p>.001 |  |  |  |
| n | 138 |  |  |  |  |
