# Supplementary material for: Functional differences between Andean oak (Quercus humboldtii Bonpl.) populations: The importance of intraspecific variation
Source: PLoS One. 2024 Mar 13;19(3):e0299645. doi: 10.1371/journal.pone.0299645 (PMC10936772; doi:10.1371/journal.pone.0299645)
Supplement: S10 Table — LT = leaf thickness; LA = leaf area; SLA = specific leaf area; LDMC = leaf dry matter content; WD = wood density; SRL = specific root length. Bold indicates significant differences (p < 0.05). (DOCX) [file pone.0299645.s011.docx]

**Supplementary material**

**S10 Table. P-values of the two one-sided statistical tests performed (lower and upper limit) to compare T_IP.IR from each population with random expectations, according to each functional trait and ontogeny stage.** LT = leaf thickness; LA = leaf area; SLA = specific leaf area; LDMC = leaf dry matter content; WD = wood density; SRL = specific root length. Bold indicates significant differences (p < 0.05).

| **Ontogeny** | **Limit** | **LT** | **LA** | **SLA** | **LDMC** | **WD** | **SRL** |
| --- | --- | --- | --- | --- | --- | --- | --- |
| Adults | inf Arcabuco | 0.201 | **0.001** | 0.189 | **0.001** | **0.020** | **0.005** |
|  | inf Chicaque | 0.147 | 0.570 | 0.937 | **0.001** | 0.041 | 0.180 |
|  | inf Encino | 0.969 | 0.842 | **0.004** | 0.312 | 0.060 | 0.840 |
|  | sup Arcabuco | 0.800 | 1.000 | 0.812 | 1.000 | 0.981 | 0.996 |
|  | sup Chicaque | 0.854 | 0.431 | 0.064 | 1.000 | 0.960 | 0.821 |
|  | sup Encino | 0.032 | 0.159 | 0.997 | 0.689 | 0.941 | 0.161 |
| Juveniles | inf Arcabuco | 0.289 | **0.011** | **0.001** | **0.002** | 0.546 | **0.033** |
|  | inf Chicaque | 0.411 | 0.312 | 0.751 | **0.029** | **0.031** | 0.051 |
|  | inf Encino | **0.043** | 0.809 | **0.012** | **0.001** | 0.417 | 0.634 |
|  | sup Arcabuco | 0.712 | 0.990 | 1.000 | 0.999 | 0.455 | 0.968 |
|  | sup Chicaque | 0.590 | 0.689 | 0.250 | 0.972 | 0.970 | 0.950 |
|  | sup Encino | 0.958 | 0.192 | 0.989 | 1.000 | 0.584 | 0.367 |
